# Consolidating metabolite identifiers to enable contextual and multi-platform metabolomics data analysis

**DOI:** 10.1186/1471-2105-11-214

**Published:** 2010-04-29

**Authors:** Henning Redestig, Miyako Kusano, Atsushi Fukushima, Fumio Matsuda, Kazuki Saito, Masanori Arita

**Affiliations:** 1 Metabolomics Research Group, RIKEN Plant Science Center, 1-7-22 Tsurumi-ku, Suehiro-cho, Yokohama, Kanagawa, 230-0045, Japan

## Abstract

**Background:**

Analysis of data from high-throughput experiments depends on the availability of well-structured data that describe the assayed biomolecules. Procedures for obtaining and organizing such meta-data on genes, transcripts and proteins have been streamlined in many data analysis packages, but are still lacking for metabolites. Chemical identifiers are notoriously incoherent, encompassing a wide range of different referencing schemes with varying scope and coverage. Online chemical databases use multiple types of identifiers in parallel but lack a common primary key for reliable database consolidation. Connecting identifiers of analytes found in experimental data with the identifiers of their parent metabolites in public databases can therefore be very laborious.

**Results:**

Here we present a strategy and a software tool for integrating metabolite identifiers from local reference libraries and public databases that do not depend on a single common primary identifier. The program constructs groups of interconnected identifiers of analytes and metabolites to obtain a local metabolite-centric SQLite database. The created database can be used to map in-house identifiers and synonyms to external resources such as the KEGG database. New identifiers can be imported and directly integrated with existing data. Queries can be performed in a flexible way, both from the command line and from the statistical programming environment R, to obtain data set tailored identifier mappings.

**Conclusions:**

Efficient cross-referencing of metabolite identifiers is a key technology for metabolomics data analysis. We provide a practical and flexible solution to this task and an open-source program, the metabolite masking tool (MetMask), available at http://metmask.sourceforge.net, that implements our ideas.

## Background

Efficient analysis of data from high-throughput experiments requires sufficient access to information about the measured biomolecules. Such data are often referred to as meta-data and provide a biological and chemical context in the form of parameters such as function, localization, and structure. Numerous applications of genomics and transcriptomics data analysis depend on the availability of meta-data, including pathway projections [1,2] and gene set enrichment analysis [3-5].

Metabolomics data analysis is no exception, and apart from the aspects of biological interpretation, meta-data are also needed for data integration. In particular, wide coverage detection of metabolites can only be achieved by combining multiple metabolomics platforms [6] such as GC-MS (gas chromatography-mass spectroscopy), CE-MS (capillary electrophoresis - MS) [7], LC-MS (liquid chromatography - MS) [8] and ^1^H-NMR [9]. However, in order to summarize multi-platform data in a consensus data set, it is crucial that the meta-data define to which metabolite each feature corresponds in a consistent and non-redundant manner.

Data on biomolecules can be found in online databases, which must be cross-referenced to allow for collation of meta-data packages for individual data sets. Creating mappings between local identifiers used in experimental data and public identifiers is a laborious process since missing, ambiguous or redundant entries are common. Identifiers are also subject to frequent changes and it is therefore clear that cross-referencing must be an automated process to enable efficient and reproducible research. For sequence-based data, several tools have been developed that can be used to automatically cross-reference public databases. The AnnBuilder R package [10] assembles and consolidates genomic information from resources such as LocusLink and the Gene Ontology. PICR [11] and PAnnBuilder [12] perform similar tasks for proteins.

Several public databases are focused on gathering information about metabolites and integrating this with data on genes and proteins. Notable examples include the Chemical Entities of Biological Interest database (ChEBI) [13], the Kyoto Encyclopedia of Genes and Genomes (KEGG) [14], the Madison Metabolomics Consortium Database (MMCD) [15] and the Human Metabolome Database (HMDB) [16]. All these databases use more than one public identifier and can therefore also be used for identifier conversion. Middleware solutions such as BioSpider [17], BioMart [18] and BridgeDb [19] are useful tools for querying these in an efficient manner. However, metabolomics data are often annotated with compound names (synonyms) of varying consistency, or in the best case, references to in-house libraries. These local identifiers can often be associated with several different molecular structures causing ambiguities and redundancies that make them very difficult to cross-reference with public identifiers. A tool that aim to solve this task has, to the best of our knowledge, not yet been reported.

There are several aspects of metabolite identifiers that make them difficult to cross-reference in an automated fashion. A major obstacle is the lack of a widespread standardized identifier. There is a multitude of different schemes for referencing chemical compounds because the best way of doing so largely depends on the purpose of the identifier. Metabolites are in general best referred to by their absolute chemical structure using e.g. InChI (IUPAC International Chemical Identifier). However, in certain circumstances it is necessary that the identifier is human readable warranting the use of chemical synonyms; on other occasions, we need to refer to a specific resource and therefore use database keys. Currently, chemical databases solve this problem by using multiple types of identifiers in parallel.

Unfortunately, with different databases relying on different identifiers, consolidation becomes very difficult [20], especially since one frequently must rely on multiple intermediate resources.

Another serious problem for data integration is that most referencing schemes are redundant in the sense that the same compound has multiple valid identifiers. Therefore, even if everyone used, e.g., PubChem IDs as suggested by Kind et al. [20], cross-referencing for the purpose of data integration may still be difficult as different identifiers do not imply different metabolites.

A related problem stems from the fact that chemical databases are geared toward annotating single, specific compounds, which is not entirely compatible with real life metabolomics data. Metabolites are often derivatized prior to separation due to analytical requirements or inexactly determined because of the limited resolution of high-throughput metabolomic platforms. Hence, the measured *analyte *does not necessarily correspond to the same chemical structure as the original *metabolite *and is therefore associated with a different identifier. These identifiers must be mapped back to their plain structure prior to biological interpretation and integration with data from other platforms. This problem becomes especially vexing since different platforms may use different analytes for the same metabolite. The abstraction between analytes and metabolites is typically only defined in platform-specific in-house libraries (e.g., [21]). Taken together, identifiers used in metabolomics are connected in a many-to-many kind of relationship, which current chemical databases do not fully support.

One approach to solve the problem of gathering meta-data for metabolomics would be to build a new database of chemical compounds including all known analytes, how they relate to parent metabolites as well as links to all relevant biological resources. Such a project, however, would be extremely resource intensive, and since different metabolomics researchers use different reference libraries and have different ambitions, it would still not solve the problem completely.

Instead, we opted for a more pragmatic strategy and designed a program that can import both in-house reference libraries and online resources and organize the identifiers by how they are interconnected. By this method, groups of compounds are formed containing both the analytes and the metabolites they refer to as well as links to the selected biological databases. Because all available identifiers are used in parallel, there is no need for any master identifier, and databases can be consolidated as long as they can be linked using any of the imported resources. The result is a metabolite-centric database, which can be used to obtain tailored metabolite meta-data in a flexible and straightforward manner.

Here, we present and discuss our strategy for reconciling metabolite identifiers across in-house libraries and public databases. Examples are given both for how to create and query a custom database as well as the types of data analysis that this technology enables. Using the provided software, MetMask (the metabolite masking tool), tailored mappings between different metabolite identifiers are easy to construct, thereby providing meta-data accessibility similar to that known from gene expression data analysis.

### Implementation

**Figure 1:**
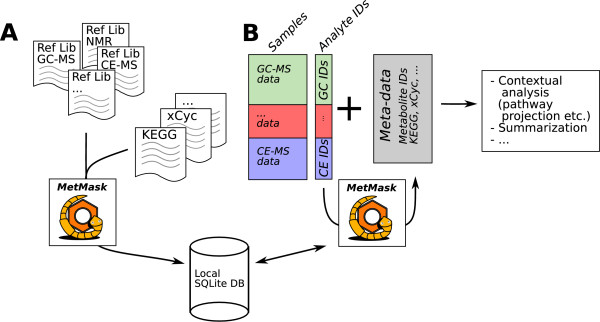
**The MetMask concept**. Metabolite identifier consolidation using MetMask. (A) A local database is created by importing public databases as well as platform specific reference libraries (Ref Lib) that list all relevant analytes and the parent metabolites to which they correspond. (B) The created database can be used to rapidly extract identifiers and meta-data to enable summarization and contextual analysis such as pathway projections.

The goal of this project was to construct a method to efficiently cross-reference different types of metabolite identifiers in order to facilitate downstream data analysis (Figure 1). Specifically, we wish to obtain a local database that associates every relevant metabolite with a group of identifiers comprising all known references to that metabolite. Because we deal with applied data analysis, two metabolites that can not be distinguished by the metabolomics platform at hand are considered the same metabolite. Every identifier group should also contain references to the platform-specific analytical derivatives, or analytes, of the associated metabolite.

These ambitions imply that the constructed groups may contain references to more than one distinct chemical structure. The goal of the constructed database is therefore clearly different from that of public chemical databases such as HMDB and PubChem [22], which gather information about specific chemical structures.

The resources that are available for constructing the desired database are chemical reference libraries and external public databases such as KEGG [14], ChEBI [13] and the *Cyc databases (e.g. EcoCyc [23], HumanCyc [24] and PlantCyc [25]). The information taken from external databases is the primary identifiers and their links to other public databases. The reference libraries are files that list the analytes that are recognized by the corresponding metabolomics platform and associate these with their parent metabolite. The listing typically uses local identifiers for the analytes and at least one publicly used identifier of the metabolite, e.g., a CAS (Chemical Abstracts Service) registry number or commonly used synonyms.

Unfortunately, there are several limitations that make these resources non-trivial to integrate with each other.

• The primary identifier that we seek, the identifier group, is not used by any resource.

- All resources may list multiple entries for the same metabolite, e.g., separate entries for alanine and L-alanine.

• There is no identifier type that is used by all entries in all resources.

• Comparing different resources, there may be errors in the sense that the same identifier can be used to refer to different metabolites.

The input can be thought of as a large network of identifiers where primary identifiers are linked to other identifiers. A straightforward way of obtaining the groups of identifiers that we seek is to combine all identifiers that are interconnected. However, the last observation above implies that this could also erroneously group strictly different metabolites. To solve this problem we designed a heuristic, which is described in the following section.

### A strategy for cross-referencing metabolites

**Figure 2:**
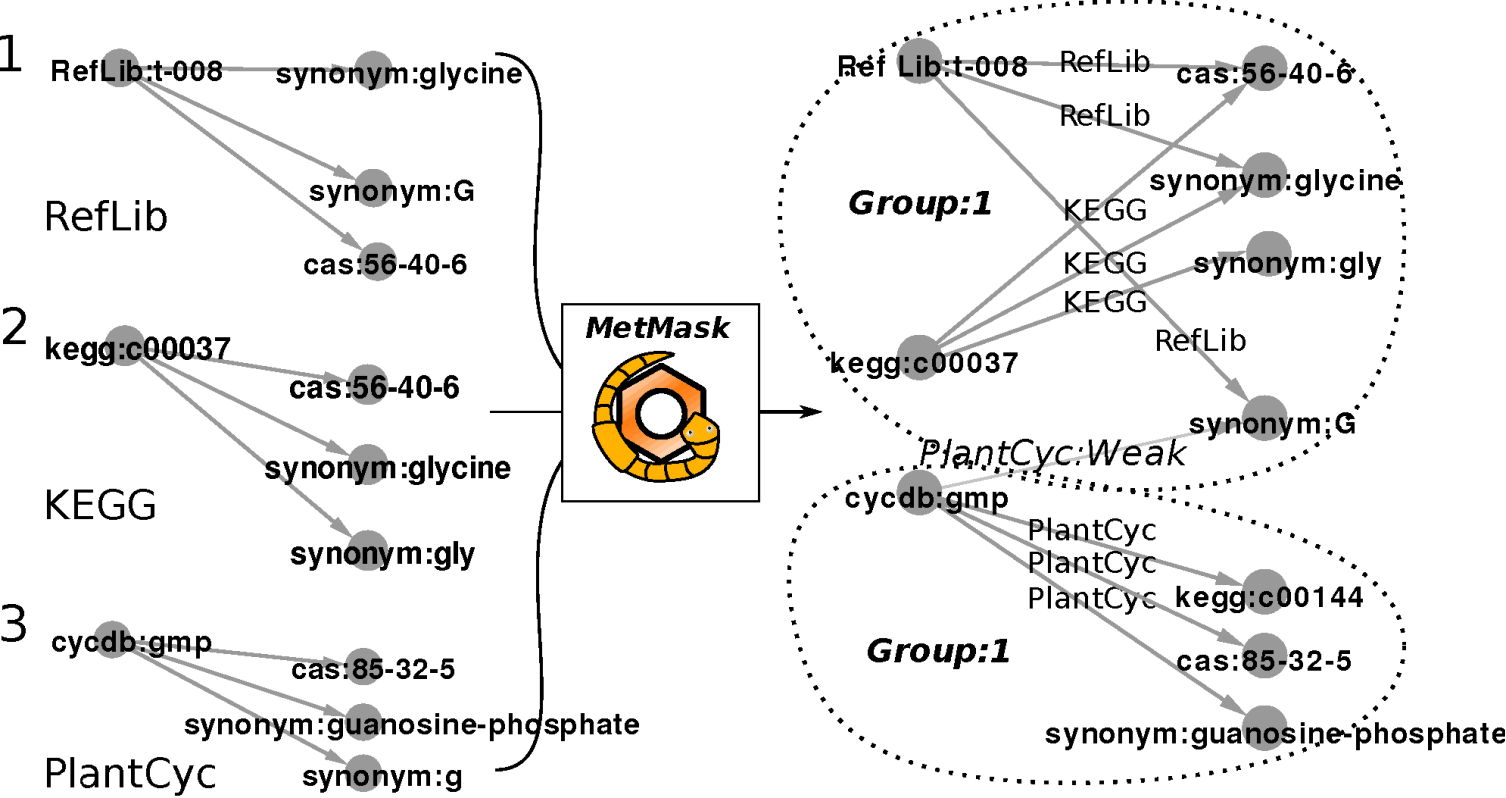
**Metabolite identifier integration**. Strategy for integrating metabolite identifiers. (1) The reference library RefLib specifies an identifier and its links. Upon import to the MetMask database, these identifiers are assigned to Group 1. (2) The KEGG entry c00037 links to both the CAS number 56-40-6 and the synonym glycine and is therefore merged to Group 1. (3) The PlantCyc entry gmp overlaps with the identifiers in Group 1 but only in a single synonym and is therefore assigned to the new identifier group, Group 2. Group 2 retains the mapping to the conflicting synonym G but has this link annotated as weak.

We reason that the primary key, the identifier group, should correspond to a group of strongly interconnected identifiers. We grow these groups incrementally as new data are imported to our database and merge groups if they overlap strongly with each other; see Figure 2 for a schematic representation of the integration strategy.

All input can be broken down into groups of identifiers that associate a primary identifier with a set of associated identifiers. Upon data import, the database that is being constructed is searched for any pre-existing identifier group that overlaps with the incoming group. If an overlapping group is found, it is tested for compatibility with the new group. If the two groups are compatible, they are merged to form a new larger group; if they are not compatible, the overlapping identifiers in the new group are annotated as 'weak' and the rest form a separate group. Weak identifiers are defined as identifiers that may be associated with more than one identifier group. The compound name C is a typical example of a weak identifier as it can refer to either cysteine or cytosine.

Because no identifiers are deleted, all original associations found in the input are also present in the resulting database and can be used to query for metabolites.

The constructed database may group different chemical structures, and therefore there is no direct way of knowing which identifiers should be connected and which should not. Hence, we resort to a rule set and define two identifier groups as compatible if,

1. They come from the same source and share a non-weak identifier.

2. They come from different sources but share at least *n *types of non-weak identifiers, where *n *is user-defined (default 2).

3. Two identifier groups are not compatible if they do not have the same chemical sum formulas (ignoring single proton differences and derivatized compounds).

The threshold in step 2 is included to cope with errors and minor ambiguities in the input. A high *n *implies that correctness is prioritized and only groups of identifiers that are very likely to refer to the same metabolite are merged. A low *n *on the other hand prioritizes non-redundancy, causing more groups to be merged. When consolidating two databases that only can be linked with a single type of identifier, this threshold should be set to one. When working with very unreliable data sources, it may instead be increased.

The resolved groups of metabolite identifiers are stored in a local database that keeps track of the source of every imported identifier, which identifier group it belongs to and if that association is annotated as weak. The identifier groups have a local primary key that allows for instantaneous conversion between different types of identifiers.

The notion of defining certain identifiers as weak also makes it possible to set entire identifier types as weak. This way, annotational identifiers such as pathway information can be imported without causing any identifier groups to be merged.

### The MetMask interface

The program interface is command line-based, making it easy to integrate into automated data analysis pipelines. Sources and binary distributions can be found at http://metmask.sourceforge.net. The project page also gives access to a web-interface, which can be used to perform identifier conversion and visualization using an example database.

The widely used statistical programming environment R [26] together with the BioConductor project [27] provides meta-data for proteins and genes but not metabolites. Therefore, we designed the metmask.db R-package which can be used in a similar way as the packages depending on the AnnotationDbi [28] framework. The metmask.db package depends on a slightly modified version of AnnotationDbi, which is available from the MetMask project page. The package can be used either with its accompanying database or with a customized database by simply replacing the database file.

The main MetMask program is platform-independent, free, open-source software implemented as a Python package. Identifiers are stored in a local SQLite [29] database and the package is distributed together with an example database including 1439 identifier groups.

### Import

Different parser modules depending on which source is being imported handle imports to the database. The parsers read different file formats but in essence all perform the same thing: collation of groups of identifiers, annotating and inserting them to the database. The currently distributed parsers are listed in Table 1. File format definitions can be found in the user manual provided as Additional file 1. The parsers modules are implemented as plug-ins making implementation of new parsers easy.

**Table 1:** Parsers.

Name	Resource	Format	Imported identifier types
simple	User provided	Comma separated text file	File specific
sdf	The NIST library	SDF chemical information file	NIST number, CAS, Synonyms, Sum formula
mpimp	MPIMP MS library	NIST MS export file	Name, KEGG, Synonyms, CAS
cycdb	Any *Cyc database	compounds.dat file	Frame ID, CAS, Synonyms, SMILES, InChI, KEGG
cyc	Any *Cyc database	compounds dump file	Synonyms, CAS, KEGG, SMILES, Sum formula, Pathway
kegg	KEGG	compounds file (local or via FTP)	KEGG ID, Synonyms, CAS, Sum formula, ChEBI, KNAp-SAcK, Pathway, PubChem SID
chebi	ChEBI	online database (SOAP)	ChEBI ID, IUPAC Name, CAS, KEGG, InChI, SMILES, Sum formula, Synonyms
metabocard	HMDB	metabocards.txt	BioCyc, CAS, ChEBI, Sum formula, HMDB, InChI, IUPAC, KEGG, Metlin, Synonym, Pub-Chem SID, PubChem CID

The currently provided parsers for importing metabolite information. File format definitions can be found in the user manual. The imported identifier types indicate the identifiers that are extracted from the source file.

Imports can be performed both comprehensively and in synchronization mode in which only identifier groups are imported that already have some overlap in the database.

The identifier types KEGG compound ID, CAS Number, KNApSAcK, CQ ID (from MMCD), PubChem Substance ID and Compound ID, InChI, Metlib ID and HMDB ID are matched with regular expressions to ensure that imported identifiers are well formatted.

Once a database has been created, minor updates that define new metabolites or add data to already existing metabolite groups may be performed by re-importing the updated source. Larger rearrangements and deletion of identifiers is not supported in the current version of MetMask and must therefore be done by rebuilding the whole database.

### Query and visualization

The MetMask database can be queried in a flexible manner, making it easy to extract both information on single entries and to do batch queries. When input is given via standard input, each line is treated as a query string, and the result is provided as standard output. Full export is also supported in which the requested identifier types are extracted for all identifier groups.

Default output is given as a comma delimited table where one row corresponds to one identifier group and each column corresponds to the queried identifier type. Multiple identifiers of the same type and group are separated by the pipe character. This type of output can be imported into spreadsheet programs or read by interpreters such as Python, Perl or R.

The associations in the constructed database are annotated with both the original source they came from and whether they are considered useful for identifying a specific metabolite or weak (only provide annotation). The identifier groups can be visualized as graphs by tracing how primary identifiers link to other identifiers in the input sources (example in Figure 2). To facilitate these visualizations, MetMask can output graphs of identifier groups as text files with one edge per row, with the source node in the first column and target node in the second column. The original source is given in the third column and the status as weak in the fourth column. This type of text file can be visualized using graph drawing software Cytoscape [30] or Rgraphviz [31].

### Provided database

MetMask is distributed with a database built for our metabolomics platform. The database is geared towards plant primary metabolism and is not meant to suit all researchers' needs but mainly to serve as an example. The database was built by importing platform specific reference libraries and synchronization with KEGG, PlantCyc (version 3.0) and ChEBI [13]. There are 1439 different identifier groups in the database representing our estimation of the total number of distinct metabolites in our reference libraries. Table 2 presents a listing of the used sources, and Table 3 shows a description of the created database.

**Table 2:** The sources for the provided database.

Name	Source	Synchronization mode	Parser
PRIMe chemical standards	In-house database	No	simple
RIKEN MS Library	http://prime.psc.riken.jp	No	riken
MPIMP MS library	Personal communication, [21]	No	mpimp
PlantCyc compounds.dat	http://www.plantcyc.org	Yes	cycdb
KEGG Compounds/Pathways	http://www.genome.jp	Yes	kegg
ChEBI	http://www.ebi.ac.uk/chebi	Yes	chebi

The sources used to build the provided database. Each source contains one or more different identifier types. Synchronization mode imports only additional data to already existing metabolite groups in the database.

**Table 3:** Statistics of the provided database.

Identifier type	Identifier name	Number of identifiers
Groups	_id	1439
PRIMe chemical standards	rlib	1287
RIKEN MS Library [33]	riken	241
Synonym	synonym	11180
Sum-formula	formula	951
CAS	cas	2416
KEGG Compounds [34]	kegg	1297
KEGG Pathway [34]	pathway	184
PubChem Compound [22]	cid	1857
PubChem Substance [35]	sid	1077
IUPAC Names	iupac	1928
SMILES	smiles	2666
InChI	inchi	1668
KNApSAcK [36]	knapsack	671
KaPPA-View [1]	kappav	261
LipidBank [37]	lipidbank	127
Lipid maps [38]	lipidmaps	178
ChEBI [13]	chebi	1177
Chemspider	chemspider	1001
MPIMP MS library [21]	mpimp	3439
PlantCyc Frame ID [25]	cycdb	495

Statistics of the provided database. The number of groups is the total number of constructed distinct metabolite groups. Each group gathers one or more identifiers of the following listed identifier types.

## Results and discussion

In order to demonstrate our approach, we look at an example data set from a CE-MS-, GC-MS-, and LC-MS-based study measuring metabolite levels in tomato fruits in two ripening stages. The data set was composed of three different data matrices, each annotated with either chemical synonyms or identifiers referring to platform-specific reference libraries. The biological aspects of the experiment are not within the scope of this study and we will only consider it as generic data set coming from three platforms were all preliminary data processing such as peak picking, deconvolution and alignment has been successfully performed.

In the following sections we use two reference libraries and public resources to create a MetMask database and then show how it can be used to cross-reference the local identifiers with public identifiers. Finally, we provide two short examples of data analysis techniques that the created identifier database enables. The results presented here are specific to how these particular libraries were constructed, but the main concept will be the same regardless of the utilized platform.

### Creating and querying an identifier database

First, we import two reference libraries consisting of comma separated text files and a NIST MS export file listing local identifiers and synonyms as well as partial links to publicly used identifiers. Using MetMask, an import into a new database called 'mydb' is performed by,

> metmask --import library-one.csv --parser simple --db mydb

> metmask --import library-two.txt --parser riken  --db mydb

This import limits the search space of the metabolites in order to obtain a stream-lined database for our libraries. When this import has been performed, we can enrich the created identifier groups with data from other sources by importing those sources in synchronization mode. Augmenting our database with data from ChEBI and KEGG is performed by,

> metmask --import chebi --synchronize --db mydb

> metmask --import kegg --synchronize   --db mydb

The two commands listed above may take up to 20 minutes but only necessary when building or updating the database.

Our experimental identifiers may be cross-referenced by querying the created database. ChEBI identifiers, KEGG IDs and synonyms for our example data set can be extracted by calling,

> metmask local-ids.txt --goal chebi, kegg, synonym -Q --db mydb

which yields

17497, c00160, glycolate|glycolic acid|hydroxyacetic acid

17794|17050, c00197|c00597, 3-phospho-dl-glycerate|3-phosphoglycerate|<cont..>

Other more complex queries such as "all CAS numbers and ChEBI identifiers for the entries associated with KEGG pathway 00500 (starch and sucrose metabolism)" are also straightforward, e.g.,

> metmask --query 00500 --table pathway --goal cas, chebi --db mydb

### Comparison with using single resources

The main, still relatively easy alternative to using MetMask for cross-referencing identifiers is to write a custom script to query a public database. In order to compare our performance with this approach, we created MetMask databases using only the example reference libraries library-one.csv, library-two.txt and the input file local-ids.txt (Additional files 2, 3 and 4) and one of the resources ChEBI, KEGG, PlantCyc, the MPIMP MS (Max-Planck Institute for Molecular Plant Physiology) library [21] and a manually curated list of chemicals which we refer to as the PRIMe (Platform for RIKEN Metabolomics) chemical standards. We then tried to convert the identifiers and compound names used in the experimental data to the public identifiers CAS, KEGG ID or InChI. These identifier types are used by all resources except the MPIMP MS library and KEGG which do not use the InChI identifier.

Table 4 lists the number of successfully converted identifiers. The result shows that although the public databases contain the sought identifiers, they cannot always be reached with the search strings found in the experimental data. However, when all databases are used together as in the MetMask strategy, identifiers are accumulated and cross-referencing becomes possible. This result shows that the MetMask approach of using multiple resources improves cross-referencing, thereby reducing the risk that metabolites get lost during identifier conversion. Of the 251 search strings, 13 were completely absent from the MetMask database. Manual examination revealed that they were either typos or rare compound names. An advantage with MetMask is that a list of such problematic identifiers can be used to create a new input file, which associates them with better known identifiers. After this input has been imported to the database, the old identifiers are directly linked to all other identifiers as well.

**Table 4:** Comparison of cross-referencing performance on the example data set.

Databases	CAS Registry number	KEGG ID	InChI	Any identifier
Only reference libraries	124	58	0	124
PlantCyc	125	82	74	125
GMD	199	146	0	202
ChEBI	124	58	54	124
KEGG	131	111	0	131
PRIMe chemical standards	168	158	166	168
All (MetMask)	235	222	231	238

Comparison of cross-referencing performance for the 251 identifiers and synonyms found in the example data set. Local reference libraries were combined with the sources listed in column Databases via MetMask and used to convert local identifiers to CAS, KEGG and InChI identifiers. The table lists the number of successfully converted identifiers. Conversion to "Any identifier" indicates the number of local identifiers that could be converted to any other type of identifier (e.g., SMILES, synonym, IUPAC name, etc.). Using all resources together, as performed in the MetMask approach, we obtain a better identifier conversion performance.

Enhanced performance in identifier conversion is, however, not the only advantage when using MetMask compared to querying single public resources. MetMask makes it possible to incorporate local identifiers to create a tailored database, something that is not supported by any online resource.

MetMask also gives a single interface for queries that facilitates and speeds up batch queries. Performing the 251 queries for the example data set takes ca. 5 seconds on a standard PC. In comparison, the identifier conversion tool BioSpider [17] use several online resources making it continuously up-to-date, but also fairly slow with a single query typically taking several minutes.

### Visualization of a group of identifiers

**Figure 3:**
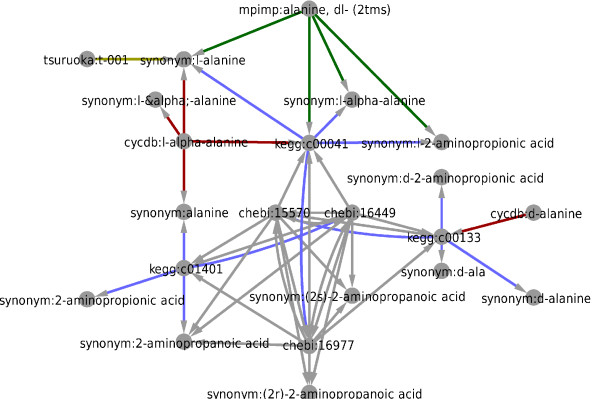
**The connection graph for the KEGG identifier "C00041"**. An excerpt of the connection graph associated with alanine. Identifiers for D-alanine and L-alanine have been merged since high-throughput metabolomics usually do not resolve optical isomers. Several of the connections are only available via intermediate steps, illustrating how complicated manual identifier conversions can be. Green edges come from the MPIMP MS library, gray edges come from ChEBI, red edges come from PlantCyc, and blue edges come from KEGG and the yellow edge come from our CE-MS library.

The associations in the example database provided with MetMask are annotated both with the original source they came from and whether they are considered to be useful for identifying a specific metabolite or are weak (only provide annotation). Identifier groups can be visualized as networks where each source connects its own master identifier to a set of externally used identifiers. An excerpt of the connection graph for alanine is shown in Figure 3. The MPIMP MS library connects alanine, -DL (2TMS) with a KEGG entry and two synonyms. As further sources were imported, more identifiers were added to the same group, making it easy to map our in-house identifier to the external resources even though those associations would require intermediate identifier types.

Note that multiple entries from the public databases ChEBI and KEGG have been merged to the same entry since the analyte alanine, DL- (2TMS) can be interpreted as any of the entries L-alanine (KEGG:C00041, ChEBI:16977), D-alanine (KEGG:C00133, ChEBI:15570) or alanine (KEGG:C01401, ChEBI:16449). This merging is an important feature because the resolution of the annotations must match that of the experimental platform and be as non-redundant as possible to avoid statistical bias toward multiply represented metabolites. Gathering all equivalent identifiers also helps to avoid inconsistencies, which may arise when the same metabolite is annotated with slightly different compound names.

Performing this kind of identifier grouping is very difficult without combining the result of queries to multiple databases.

To avoid creating connections between different metabolites we resorted to a heuristic rule-based approach. Our capability to detect erroneous input is currently limited to ensuring good overlap and matching sum formulas between identifier groups before merging them. Therefore, our accuracy depends largely on correct input. After importing new resources to the database it is recommendable to inspect the output manually to confirm the result. If mistakes are discovered, the graph visualization capability of MetMask provides a way to track down the source of the errors. False associations can then be dropped from the database to avoid future errors. The main alternative to MetMask, writing custom scripts, is not less error-prone, particularly since chemical databases tend to be sparsely connected requiring the use of intermediate identifiers.

### MetMask facilitates multi-platform metabolomics and contextual data analysis

Identifier unification plays an important role if one combines multiple analytical platforms to obtain better coverage of the metabolome. Different platforms may use different reference libraries, which results in data sets with mixed types of identifiers. In order to obtain a consensus, non-redundant data set, it is crucial that metabolite identifiers are used in a consistent manner. Efficient identifier management is therefore a key technology for multi-platform metabolomics. Current middleware solutions exemplified by BioMart [18] and BioSpider [17] provide efficient access to online resources but do not resolve any ambiguities or redundancies that they imply.

**Figure 4:**
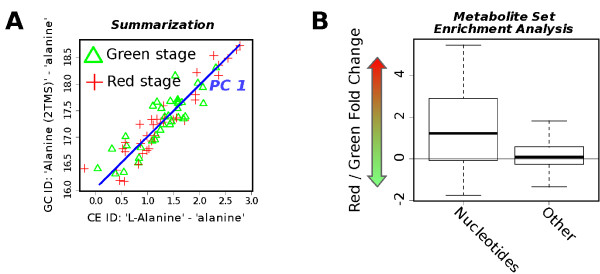
**Obtaining a consensus metabolite feature**. Examples of enabled technologies. (A) MetMask makes it easy to cross-reference identifiers used in different metabolomics platforms. Once this has been done, features representing the same metabolite can be summarized using, e.g., principal component analysis to obtain a consensus data set. Here, an example is shown where the features from CE-MS and GC-MS that represent alanine are replaced by PC_1_. (B) MetMask can link unified identifiers to annotation databases such as PlantCyc, thereby allowing for contextual interpretation such as metabolite set enrichment analysis. The boxplot shows that the log fold changes between red and green tomatoes are higher among the nucleotide synthesis related metabolites than the other metabolites.

After the identifiers in the example data set are unified using MetMask, all analytes that correspond to the same metabolite can be extracted and summarized by, e.g., replacing them with their first principal component (PC). In Figure 4A, the alanine features from CE-MS and GC-MS are replaced by their first principal component (PC). This procedure can then be repeated for all duplicated metabolites until all features are unique. Without proper identifier integration, this task would require manual intervention - an unfeasible process when working with wide coverage metabolomics data. The obtained consensus data set has the advantage over the original data that it is not biased towards the number of features that represent each metabolite. Following data analysis thereby become easier to interpret and false positives due to multiply represented metabolites can be avoided.

MetMask can also link to databases with biological annotation and thereby facilitate biological interpretation. In Figure 4B, the fold-changes between red and green ripening stages were sorted in to their metabolite classes as suggested by PlantCyc. In a manner analogous to the gene set enrichment analysis (GSEA) [3] where sets of genes are tested for association with a particular response variable, we can perform metabolite set enrichment analysis (MSEA). Using the Kolmogorov-Smirnov test (KS), we test each class of metabolites to examine if the distribution of fold-changes within the class differs from that of the metabolites outside the class. Here, we found that metabolites related to nucleotide/nucleoside synthesis, e.g., ribose, uridine, guanine and adenosine, have been up-regulated when comparing green tomatoes to red tomatoes (KS test *P *= 0.0002, false discovery rate = 0.006). This observation is supported by Carrari et al.'s (2006) finding that transcripts for nucleotide conversion enzymes are strongly affected during tomato development [32].

## Conclusion

Cross-referencing metabolite identifiers and gathering meta-data are essential technologies for metabolomics data analysis. There are several public databases that contain information on metabolites, but linking these data with the local identifiers and compound names often used in experimental data sets has been very difficult. Here we presented a novel strategy for creating a database that gathers and organizes information about metabolites from both in-house reference libraries and external resources. Our approach uses multiple identifier types in parallel when consolidating databases, thereby avoiding the problem of lacking a widely used identifier scheme. Issues with redundant and missing entries are addressed by importing multiple resources to create a unified identifier database.

The MetMask tool provides an implementation of our ideas and can be used to create tailored metabolite mappings with minimum user effort. Efficient handling of identifiers enables data summarization and biological interpretation via contextual analysis such as pathway projections.

## Availability and requirements

**Project name: **metmask

**Project home page: **http://metmask.sourceforge.net

**Operating systems: **Platform independent (tested on Windows XP and Ubuntu Linux)

**Programming language: **Python

**Other requirements: **None

**License: **GNU GPL

**Any restrictions to use by non-academics: **None

## Authors' contributions

HR initiated the project, designed and implemented the program and wrote the manuscript. MK wrote the manuscript, provided GC-MS data and evaluated the program. AF evaluated and tested the program and wrote the manuscript. FM provided LC-MS data, curated our library of reference compounds and contributed to the graph based visualization approach. KS and MA supervised the project.

All authors read and approved the final version.

## Supplementary Material

Additional file 1 **User manual for MetMask**. Instructions for installation and usage. Also available by the project webpage. Click here for file

Additional file 2 **Example reference library - CSV**. The comma separated text file based reference library used in the demonstration section. Click here for file

Additional file 3 **Example reference library - NIST**. The NIST MS data export based reference library used in the demonstration section. Click here for file

Additional file 4 **Example input**. The file with local identifiers used in the demonstration section. Click here for file
