# Supplementary material for: Consolidating metabolite identifiers to enable contextual and multi-platform metabolomics data analysis
Source: BMC Bioinformatics. 2010 Apr 29;11:214. doi: 10.1186/1471-2105-11-214 (PMC2879285; doi:10.1186/1471-2105-11-214)
Supplement: Additional file 1 — User manual for MetMask. Instructions for installation and usage. Also available by the project webpage. [file 1471-2105-11-214-S1.PDF]

Masking metabolite identifiers - A user manual for MetMask

Henning Redestig

05 November 2009

Contents

1 Introduction **2**
- 1.1 Metmask . . . 2
  - 1.1.1 Features . . . 2
  - 1.1.2 What metmask is not . . . 3
  - 1.1.3 Why metmask? . . . 3

2 Installation **3**
- 2.1 Linux . . . 3
- 2.2 Windows . . . 4

3 Configuration **4**

4 Usage **5**
- 4.1 Examples . . . 7
  - 4.1.1 Import . . . 7
  - 4.1.2 Query . . . 8
- 4.2 Special usages . . . 9

5 Parsers and file formats **9**
- 5.1 Import formats . . . 9
- 5.2 Export formats . . . 10

6 Implementation **10**
- 6.1 Glossary . . . 11
- 6.2 Strategy . . . 11
  - 6.2.1 Merging masks . . . 11
  - 6.2.2 Confidence code . . . 12

7 Additional remarks **12**
- 7.1 Version numbers . . . 12
- 7.2 Caveats . . . 12
- 7.3 Parsers . . . 12
  - 7.3.1 Write your own parser . . . 12

8 R-integration **13**
8.1 Via the commandline 13
8.2 Via the metmask.db package 14

1 Introduction

Obtaining meta-information (biological annotation etc) for metabolomics data can be a quite tedious process involving scripting, cross-referencing and hand curating multiple online databases. Reasons for this include :

- Identifiers of metabolites used in raw data often is not easily convertible to the indentifiers used by online databases.
- Online databases often do not share any single primary keys.
- Databases may list multiple entries for the same metabolite.
- No single identifier type is used by all resources.
- Comparing different resources, there may be errors in the sense that same identifier can be used to refer to different metabolites.

These problems makes sharing, managing and analysing metabolomics data tedious since it is difficult to keep a machine readable list of the different ways to reference metabolites.

1.1 Metmask

This is where the metmask tool comes into the picture by facilitating the process of mapping identifier names, including identifiers of **analytes**, to other known identifiers. The process of grouping many identifiers to a single entity is here called to **mask** the identifiers. The group of identifier is correspodingly called a **mask** Currently metmask can do the following:

1.1.1 Features

- Incorporate an arbitrary list with any kind of identifier and keep track of which identifier is associated with which by assigning them to internal mask identifiers.
  - Keep track of the connection information, i.e. which source was used for which connection.
  - Annotate connections as *weak* or *non-weak* depending on if they are ambiguous or not.
  - Fusing different masks if input data implies that the masks are the same.
- Import widely used databases such as the main NIST library, the KEGG and PlantCyc compounds files.
- Query the database and extract one or all related identifiers for any known identifier.

- Query PubChem and KEGG for identfiers that are not in the local database and save any new associations.
- Output in different formats, including graphs which can easily be visualized using e.g. cytoscape or R (with Rgraphviz).

1.1.2 What metmask is not

Metmask in its current form does not:

- Provide a way to identify metabolites based on their Mass spectra, chromatographical retention indexes or similar. Metmask can however associate ambiguous information (such as mass forumlae) and provides a way to search for these.
- Ensure that the identifiers mapped to by the database is correct in terms of what the original creators of the identifiers intended. Metmask only knows what is being imported into the database and it holds that if you put garbage in you get garbage out.

1.1.3 Why metmask?

A great advantage of metmask compared to other databases [1] such as more comprehensive databases such as PubChem is that

- it is easy to tailor to only load the desired sources in the database so that it matches the desired platform. For e.g. GC-MS metabolomics data, this would imply that all stereo-isomers of one metabolite should be behind the same mask since stereo-isomers usually are not resolved.
- comparing to online queries metmask is very fast
- combining different databases is easy whereas online databases may be lacking the source of interest (e.g. one cannot directly query for AraCyc using PubChem but this is easily done in metmask).
  - connections which requires intermediate identifiers is also straight-forward in metmask.

A word of caution, metmask assumes certain basic skills working with a shell. If you do wish to use metmask but feel that you need a graphical user environment, then please post a message on the user forum at http://metmask.sourceforge.net.

2 Installation

2.1 Linux

Download metmask-<x.y.z>.tar.gz and

1. Unpack with

[1]

```
tar -zxvf metmask-<x.y.z>.tar.gz
```

2. Go to created directory

```
cd metmask-<x.y.z>
```

3. install either as root

```
sudo python setup.py install
```

or locally

```
python setup.py install --home=<dir>
```

4. in case you used the –home switch, tell python where you installed the package e.g.

```
export PYTHONPATH=<dir>/lib/python
```

2.2 Windows

Download metmask-<x.y.z>.exe and execute it. Open the windows shell via the start menu (run command "cmd"). Note that metmask is currently commandline only so you need to type the commands.

3 Configuration

When metmask is first executed it creates the file "~/.metmask.cfg" which specifies the defaults values for the options listed above. Options given explicitly over-rides the configuration file.

The package comes with a default database is the one queried automatically. If you install the package as adminisitrator/root you will not have write-access to this database and will therefore not be able to edit the database – only query it. To get your own database, just copy the default database (its location will be in the configuration file after you first run the program or shown when trying to execute metmask) to wherever you have read/write access and edit the configuration file accordingly:

```
...
[general]
db = <path to your read/write accessible database>
...
```

Further configuration options are shown below.

Table 1: Configuration options defined in the file .metmask.cfg

Section	Option	Meaning
Simple	na	A regexp for strings to be considered missing values
	sep1	Major separator, for separating identifier types
	sep2	Minor separator, for separating identifiers
General	confidence	Default confidence code
	goal	Default identifier type to search for
	db	Path to database
	kegg	URL to the KEGG compounds file
	cyc	URL to the PlantCyc compounds database
	ask	Should merging of masks be done interactively or not
	minoverlap	Number of *types* of identifiers must overlap between two groups of metabolites

4 Usage

The program is interfaced using the script called metmask. To invoke it just execute "metmask" from the command-line. The default action is to read identifiers from standard input, guess what kind of identifier it is and then query the local database for that identifier. The following options are available:

```
metmask --version

 This is metmask v0.5.1

metmask --help

Usage: metmask [X] [options]
X represents a stream of identifiers from STDIN
or a single identifier if -a is set or a file with
identifiers, one per line. If X is not provided one
or more of the options below must be set.

Options:
  --version             show program's version number and exit
  -h, --help            show this help message and exit
  -a IDENTIFIER, -q IDENTIFIER, --query=IDENTIFIER, --as-is-id=IDENTIFIER
                        A single  identifier to query the data base for to be
                        interpreted as is. Also searches for weak (ambiguous)
                        identifiers is the universal argument is set
  -w, --wild            Input query may contain wild cards ('_' matches any
                        single character and '%' matches any sequence of
                        characters).
  -t TABLE, --table=TABLE
                        Identifier is of type TABLE (i.e. identifier type).
```

```
                      Metmask tries to guess which table is meant in case
                      unspecified. Multiple tables can be given comma
                      separated.
-g TABLE, --goal=TABLE
                      Fetch goal identifier of TABLE or use 'ALL' to get all
                      entries. Multiple tables can be given pipe separated.
-d PATH, --db=PATH    Use database located at PATH
-s, --stats           Print some statistics of the current database. Print
                      more stats if the universal argument is set.
-v, --verbose, --debug
                      Be more verbose, print debug information
-i FILE, --import=FILE
                      Populate database using this file. For importing the
                      KEGG compounds file or the PlantCyc compounds file,
                      FILE can also be one of the keywords 'cyc' or 'kegg'
                      in order to use the ftp provided files. Local files
                      have precedence. Universal argument: Never ask before
                      merging on input, guess what to do
-n NAME, --name=NAME  Each import is given a name, usually this equals the
                      filename or name of external database but can be set
                      explicitly with this option
-c CODE, --confidence-code=CODE
                      The confidence code of the data to import
-p PARSER, --parser=PARSER
                      Use this parser to populate the database
-o OUTPUT, --output=OUTPUT
                      Output modes, flat: comma delimited output, mask: a
                      text represenation of all info in the mask, graph: a
                      connection graph to use for visualization in e.g.
                      cytoscape.
-M MASTER, --master=MASTER
                      Master identifier for this import
-m, --merge-interactively
                      If set, ask about merging masks upon insert.
-r MMID, --remove=MMID
                      Drop mask with this mmid. Can not be undone!
                      Universal argument: Do not ask about deletions before
-x TABLES, --export=TABLES
                      Export all information in the provided tables (comma
                      separated, potentially quoted). Use the keyword ALL
                      for exporting all known tables.
-u, -F, --universal   Universal argument. If set, causes some arguments to
                      act differently.
-f, --first           Only return one identifier of each queried table.
                      Suppress printing multiple identfiers.
-e, --external        Try to query PubChem and KEGG if no suitable match
                      could be found. Universal argument: Save retrieved
```

```
                          information in the local database
  -1, --one-hit           Only return the first hit  from the database
  -Q, --no-quote          Suppress addition of quotes to output
  -S, --synchronize       Upon import, minimize the creation of new masks,
                          instead just populate the existing ones and ignore all
                          other input
```

4.1 Examples

4.1.1 Import

To import the KEGG compounds file directly from the KEGG FTP server:

```
metmask -i kegg
```

To import a comma delimited file, test.csv, with identifiers structured as

```
myId,synonym,weak:type
a1,alanine,amino-acid
a2,ala,amino-acid
a1,ala,amino-acid
a3,sucrose,sugar
a4,lysine,amino-acid
```

we call

```
metmask --import test.csv --parser simple --master myId --db /tmp/test-db
```

The first line in a test.csv names the types of identifiers. These will afterwards be possible query using the -t switch e.g.

```
metmask --query a1 --table myId --goal synonym --db /tmp/test-db
```

```
'"alanine"|"ala"'
```

The input comma delimited file should be structured as:

```
tablename1 , <confidence code>:tablename2 , tablename3
table1-id1 , table2-id1                   , table3-id1|table3-id2|table3-id3
table1-id1 , table2-id1                   , table3-id1|table3-id2|table3-id3
...
```

and is in the example above read by the parser "simple". Note that the confidence code can be used to specify whether a table is "weak" or not. In the example, test.csv, type is used to set a metabolite type but this type is not used to group metabolites, only to annotate them. See *Query for further details.

4.1.2 Query

To query the database for everything it knows about alanine::

```
metmask --query alanine --goal ALL --output mask -d /tmp/test-db

o-o-o-o:
mask:

myId:
a1 test.csv 3 test.csv 3
a2 test.csv 3
synonym:
alanine test.csv 3
ala test.csv 3 test.csv 3
preferred:
2:ala test.csv 0
```

or to get all metabolites we specified as type "amino-acid" in a format that is easy to import to other programs.

```
metmask --query amino-acid --table type --goal myId,synonym -d /tmp/test-db

 '"a1"|"a2"','"alanine"|"ala"'
 '"a4"','"lysine"'
```

Note that the input has been masked so that "a2" also is associated with "alanine" even though this was never explicitly specified in the input.

The import also specified a 'master' identifier which is interpreted as the source for connections. In this case, each line in test.csv is therefore read as myId *connects* to synonym. When a master identifier has been set, we can produce graph like output:

```
metmask --query a1 --table myId --goal ALL --output graph --db /tmp/test-db -u

 myId:a1       synonym:alanine test.csv        False
 myId:a1       synonym:ala     test.csv        False
 myId:a1       type:amino-acid test.csv        True
 myId:a2       synonym:ala     test.csv        False
 myId:a2       type:amino-acid test.csv        True
```

which gives the previous list (which can be imported to Cytoscape for visualization, simply click import from file and set the first column as source node and second column as target node.). The columns are Node 1, Node 2, Source of edge and an indicator for whether the connection should be considered "weak" or not. The "-u" switch tells metmask to also output weak identifiers.

If you have a file of identifiers that you want to query the database for KEGG identifiers you can simply use standared shell re-direction and query as:

```
  cat myFileWithIdentifiers | metmask -g kegg
```

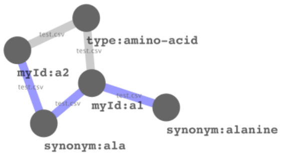

Figure 1: The connection graph for the example file test.csv as visualized by Cytoscape.

4.2 Special usages

A problem one sometimes faces is that used identifiers (e.g. synonyms) for is that identifiers are difficult to use in graphical representations such as plots. Manual conversion on a 'plot-per-plot basis' is of course not desirable and for this purpose one can use the special metmask table 'preferred'. Each mask is given exactly on preferred identifier upon import (the first non-empty identifier if it is not set explicitly). One way to fix the synonyms (assuming they are already known to the database) would be to export the current preferred labels:

```
cat mySynonyms | metmask -g preferred > myPreferred
```

then merge to get a new input:

```
paste mySynonyms myPreferred > myInput
echo -e "synonym\tpreferred" | cat - myInput
```

then manually edit "myInput" to set the preferred labels as desired and do:

```
metmask -i myInput -p simple
```

now you can query those any identifiers associated with the original synonyms and be sure to always get your desired preferred labels.

5 Parsers and file formats

5.1 Import formats

The parsers are modules that can read specific file formats, extract the important data and commit it to the database. The parsers that are included in the main distribution are listed in Table 2 and 3.

The CSV file is structured so that the first line is header naming the types of identifiers that are listed below. The header my also specify if all indentifiers below should be imported as weak eg

```
mylocalid,cas,synonym,weak:formula
```

for a file specifiying links between mylocalid a CAS registry number, synonyms and sum formula (which should be imported as weak). Following lines list the identifiers. Multiple identifiers for the same identifier type are separated by the pipe "|" character. Identifiers that contain comma must be surrounded by quotes (nested quotes are allowed) eg:

```
id-001,"'50-40-1'|'50-43-2'","1-2,non-existing-compound",C6H6O6
```

Table 2: Available parsers. Name is the name of the parser module, Tables are the types of identifiers that are extracted and imported from the resource, Weak tables are types of identifiers that are imported as weak tables.

Name	Type of input	Tables	Weak tables
chebi	ChEBI	chebi, iupac, cas. kegg, inchi, smiles	synonym, formula
cycdb	compounds.dat	cycdb, cas, inchi, kegg	synonym, smiles
cyc	compounds dump file	cas, kegg, smiles	formula, synonym, cycpath, smiles
kegg	kegg compounds	kegg, synonym, cas, chebi, knapsack, sid	formula, pathway
mpimp	NIST MS export file	kegg, synonym, cas, mpimp	
riken	NIST MS export file	riken, cas, kegg, synonym	formula, smiles
sdf	SDF	nist, cas, synonym	formula
simple	CSV file	depends on input	

Table 3: File formats

File	Format
ChEBI	Online database, read by the ChEBI SOAP service
compounds.dat	http://bioinformatics.ai.sri.com/ptools/flatfile-format.html#compounds.dat
compounds dump file	ftp://ftp.plantcyc.org/Pathways/README.txt
kegg compounds	ftp://ftp.genome.jp/pub/kegg/ligand/README
NIST MS export file	http://chemdata.nist.gov/mass-spc/amdis/AMDIS.pdf
SDF [2]	http://www.pharmainformatic.com/html/sd-format.html.
CSV file	Comma separated table

5.2 Export formats

Output is done in three different formats.

flat Text file, where each line represents one identfifier group, identifier types are by default delimited by comma and individual identifiers delimited by the pipe character. Double quotes surround each identifier type and single quotes surround each identifier.

graph A tab delimited file with four columns and one line per edge in the output graph. First colum is the source node, second column the target node, third column indicate the source of the edge and fourth column indicate if link is weak or not.

mask An overview text representation of the entire identifier group. One identifier per line and every identifier types is preceeded by the name of that identifier type as a header.

6 Implementation

This section is not directly necessary to read in order to use the program.

6.1 Glossary

Before describing the current implementation it is necessary to clarify some concepts:

mask A group of identifiers that all are associated with the same biological compound.

table A type of identifier such as CAS number or KEGG ID.

identifier A specific identifier such as the KEGG ID C00001.

analyte A compound that is identified by the chromatographic method at hand.

metabolite A chemical compound that can be found **in vivo**

preferred A special table that holds excatly one string for each mask. This string is meant to provide a human readable name for each mask.

weak An "identifier" which can not be used as metabolite identifier.

6.2 Strategy

Metmask uses a local sqlite3 database to which it can import information from various user provided re-sources. Ideally, the first import to the database should be a curated list of identifiers listing masks that definitely map to different metabolites. These are then used as a seed data-set to which one can add more identifiers by importing the desired sources.

Every association is annotated with two attributes. Where it came from, i.e. its source and its confidence code. The source is kept so that, upon later exports it is easy to see the chain of evidence. The confidence code is a bit more complicated and is used to determine which masks is allowed to be merged with which, see Merging masks.

6.2.1 Merging masks

As an import proceeds metmask ensures that a single identifier only maps to a single mask. If one tries to do an import that matches an already existing mask a conflict arises and the following alternatives are available to resolve the problem:

- Merge the new information to the exiting mask.
- Annotate the overlapping information as 'weak' and create a new mask.

To minimize errors the following rule-set is used to judge if two masks should be merged or not.

1. Two masks coming from the same source are compatible if they share a non-weak identifier.
2. Two masks coming from different sources are compatible if they share at least n types of identifiers, where n is user-defined.
3. Two masks are not compatible if they are associated with non-equal sum-formulae (ignoring single protons).
4. Two masks are not compatible if both carry an indentifiers annotated with 'nevermerge'.

6.2.2 Confidence code

The confidence code is an code that which defines whether masks with those confidence codes are allowed to be merged or not. The code is either an arbitrary string chosen upon import such as "good" or "bad" depending on the quality of the source or one of the reserved codes:

nevermerge Two masks that carry identifiers tagged with "nevermerge" will, as the name suggests never be merged.

weak An indentifier tagged as "weak" does not count when counting overlap between two masks. All identifier are unique, but when adding a mask carrying e.g. the identifier G will not be added to the Glycine mask which also carries G if G was tagged as "weak".

7 Additional remarks

7.1 Version numbers

Metmask version numbers are on the form x.y.z. For smaller changes z is increased. Changes to the database structure and the tables that are expected to be found in it always include an increase to y. An increased value of x indicate a stable version which is to be considered feature complete.

7.2 Caveats

The following is a list of irregular behaviour of metmask.

- Metmask does not allow the pipe character "|" in identifiers. Any pipes are silently discarded upon import.
- Metmask does not perform any comprehensive integrity checks of the input, if a incorrect parser is specified, the input might corrupt the database.

7.3 Parsers

If you have type of source file which can not easily be converted to the format above you need to either Write your own parser or ask for help to do so. It is fairly easy to do so both strategies will probably be successful. Please contribute any new parsers to the metmask project.

7.3.1 Write your own parser

The parsers are managed in a plug-in like system. See the source package metmask/parse for examples, by putting a new parser and naming it "_newparser.py" in that directory, it immediately ready to use. See following example for how a parser should be structured.

```
from metmask.mask import mask
import metmask.parse
from main import fileFormatError
# function for fixing a string to a vector considering quotations etc
from main import fixLine
```

```
class parser :
    """ < your documentation >
    """

    # constructor
    def __init__ (self, parent) :
        # parent is the 'main' class of parser which handles file
        # reading and communication with the metmask database

        # make sure to set the table that you use, these are used to
        # create corresponding tables in the database e.g.
        parent.tables = ['cas', 'mylocalid']
        self.parent = parent

    # the function that does the actual reading
    def process (self) :
        parent = self.parent
        # get a line of input
        ll = parent.getLine()
        while ll :
            # create a mask object
            un = mask({}, parent.mm.idpatterns)
            # append the information to the mask (see
            # documentation of the mask object)
            un.append(<table Type>, ll[0], ll[1], ll[2])
            # send the mask to the database
            parent.setMask(un)
            # get a new line
            ll = parent.getLine()
```

8 R-integration

8.1 Via the commandline

Since Metmask is a commandline tool it is easy to use it from R via the use of R's "system" function. An example for checking what CAS numbers and synonyms are (non-weakly) connected to the KEGG identifier C00002 could be:

```
system("metmask -a c00002 -g cas,synonym -Q", intern=TRUE)
```

"56-65-5" "42373-41-1" "987-65-5" "adenosine 5'-triphosphate" "adenosine-5'-triphosphate dipotassium sa

obviously, this will only work if metmask in your executable path.

8.2 Via the metmask.db package

One can also use the metmask.db package provide for to interact with the metmask database. Download and install the “special non-official version” of bioconductors AnnotationDbi (version 6.6.6) available via the metmask project page. Also download and install the metmask.db package. In R, the provided database can be queried just as the other AnnotationDbi packages (see vignettes for both AnnotationDbi and metmask.db) e.g to query for the KEGG identifiers associated with alanine:

```
library(metmask.db)
qmetmask("alanine", "kegg")
```

“c00041”
“c00133”
“c01401”

$[1]$ There are databases that try to map metabolites comprehensively, eg biospider but although they are very useful, they are much too slow to be used for day-to-day metabolite identifier mapping

$[2]$ For SDF files, identifier type is indicated by e.g. > <CASNO> following a listing of the associated identifiers. Entries are separated by four dollar signs.
